# Nanometer-scale distribution of PD-1 in the melanoma tumor microenvironment

**DOI:** 10.29328/journal.jro.1001048

**Published:** 2023-05-10

**Authors:** Colin J Comerci, Dannielle G McCarthy, Mehdi Nosrati, Kevin B Kim, Mohammed Kashani-Sabet, WE Moerner, Stanley P Leong

**Affiliations:** 1 Department of Chemistry, Stanford University, Stanford, CA, 94305, USA; 2 Biophysics Program, Stanford University, Stanford, CA, 94305, USA; 3 Division of Biological Sciences, University of California San Diego, La Jolla, CA, 92093, USA; 4 Chan-Zuckerberg Initiative, 801 Jefferson Ave, Redwood City, CA, 94063, USA; 5 Center for Melanoma Research and Treatment, California Pacific Medical Center, Research Institute, San Francisco, CA, 94107, USA.

**Keywords:** Super-resolution fluorescence microscopy, Tumor microenvironment, Melanoma, Immune checkpoint inhibitor, PD-1

## Abstract

The nanometer-scale spatial organization of immune receptors plays a role in cell activation and suppression. While the connection between this spatial organization and cell signaling events is emerging from cell culture experiments, how these results translate to more physiologically relevant settings like the tumor microenvironment remains poorly understood due to the challenges of high-resolution imaging *in vivo*. Here we perform super-resolution immunofluorescence microscopy of human melanoma tissue sections to examine the spatial organization of the immune checkpoint inhibitor programmed cell death 1 (PD-1). We show that PD-1 exhibits a variety of organizations ranging from nanometer-scale clusters to more uniform membrane labeling. Our results demonstrate the capability of super-resolution imaging to examine the spatial organization of immune checkpoint markers in the tumor microenvironment, suggesting a future direction for both clinical and immunology research.

## Introduction

Immune checkpoint inhibitors (ICIs) have revolutionized cancer treatment over the past decade. By targeting inhibitory receptors on immune cells, they work to bolster the patient’s own immune system against cancer. For instance, a variety of antibodies targeting programmed cell death 1 (PD-1), or its ligand PD-1 ligand 1 (PD-L1), have been approved as therapies for a variety of cancers, including melanoma. Unfortunately, while these treatments can be transformative for a subset of patients, a majority of patients receive little benefit from ICIs. Thus, a better understanding of PD-1, particularly in the tumor microenvironment, may provide insight into maximizing immunotherapy effectiveness, avoiding therapy-related toxicity, and ultimately guide the decision of which therapy to provide for individual patients [1,2].

At the same time that ICIs were transforming oncology, super-resolution fluorescence microscopy revolutionized biology research. While fluorescence microscopy is fundamentally limited in resolution to ~ 250 nm by the diffraction limit of visible light, a series of techniques called super-resolution microscopy have pushed beyond that resolution limit. These techniques allow imaging with resolution far beyond the diffraction limit, revealing the distribution and molecular dynamics of various subcellular structures [3,4]. While these techniques have largely been applied to cell culture samples, they have also been successfully used in mouse and human tissue sections [5–7], as well as live animal models [8].

In the field of immunology, super-resolution microscopy enabled research into the structure and assembly of immune receptors and the immunological synapse [9,10]. Imaging of cultured immune cells demonstrates that the spatial organization of various immune receptors can reveal ligand binding and early signaling events [11]. It is now appreciated that immune cell activation relies on these changes in receptor organization, at least in cell culture systems, but translating this understanding to a more physiological environment, such as the tumor microenvironment, has proven challenging.

Here, we apply super-resolution microscopy to human melanoma tissue sections to study the nanometer-scale distribution of PD-1 in the tumor microenvironment. We find that PD-1 can form ~ 200 nm clusters, although there is a wide variation in PD-1 cluster size, even within a single patient sample. Our results demonstrate the applicability of super-resolution imaging to immune checkpoint markers in the tumor microenvironment and suggest exciting future directions for both clinical and basic biology research.

## Materials and methods

### Ethics statement

Tissue collection to support this study was performed under the auspices of a protocol approved by the Sutter Health Institutional Review Board and informed consent was obtained from each patient whose tissue was included in the analysis.

### Tissue section preparation

**Figure 1:**
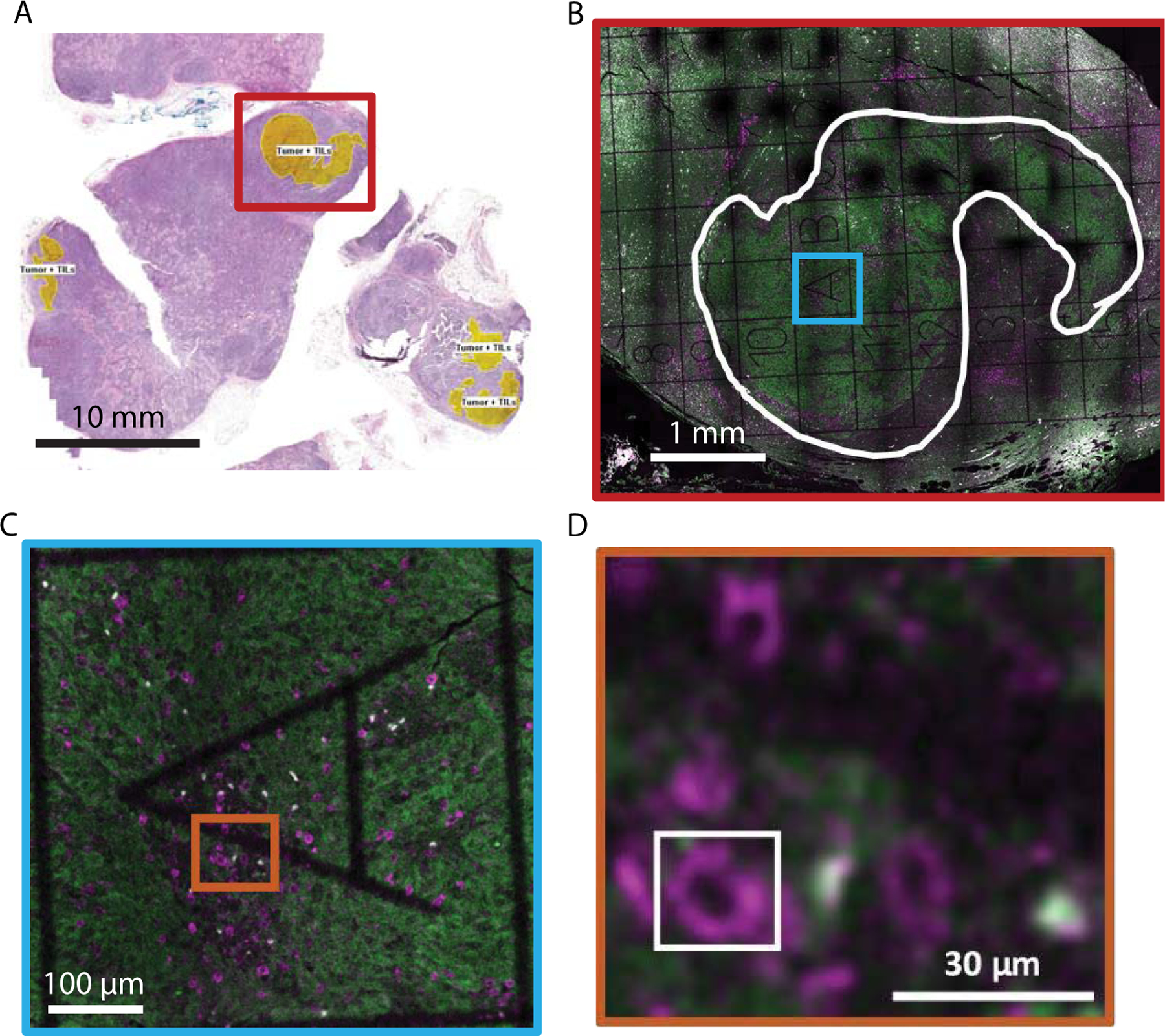
Immunofluorescence labeling and tumor microenvironment identification in paraffin-embedded melanoma cancer tissue. (A) Image of Hematoxylin-Eosin stained tissue section. Yellow regions indicate tumor regions with Tumor-Infiltrating Lymphocytes (TILs). The red box roughly marks the region imaged in (B). (B-D) Representative diffraction-limited confocal fluorescence microscopy images of an adjacent tissue section to (A). Immunofluorescence labeling is used to show PD-1 (magenta) and melanoma (green). A gridded coverslip is used to aid in the identification of regions of interest for high-resolution imaging. In (B), the white curve outline denotes the tumor area. (C) and (D) show zooms of the colored boxes in (B) and (C), respectively. The white box in (D) outlines a PD-1+ cell, showing the region typically imaged using super-resolution microscopy.

5 μm thick serial sections from Formalin-Fixed Paraffin-Embedded (FFPE) tissue were obtained from two patients with a melanoma primary tumor and matched lymph node metastasis. The samples selected were identified as PD-L1 positive by immunohistochemical analysis performed in a commercial laboratory (and found to have > 30% PD-L1 expression in the metastatic tumor). Sections from the block underwent H&E histological staining, with regions of tumors, immune cells, and Tumor-Infiltrating Lymphocytes (TILs) identified by a pathologist (Figure 1A). These histological images were used to identify regions of interest for super-resolution fluorescence microscopy.

Immunostaining was carried out on adjacent tissue sections. Careful sample preparation was essential to reduce fluorescent background as much as possible to enable high-sensitivity fluorescence imaging. Samples were dewaxed in xylene (2 × 2 min) and then rehydrated in diminishing ethanol solutions before immersing in 1X Tris-buffered saline (TBS). Samples underwent heat-induced epitope retrieval in sodium citrate buffer (11 mM trisodium citrate, 0.05% Tween 20, pH 6.0) at 100 °C for 10 minutes, followed by washing in nanopore water at room temperature (RT), before returning slides to TBS. After further permeabilizing the sample in a 0.3% (v/v) Triton x-100 solution (2 × 15 min), samples were blocked in 10% (v/v) goat serum with 3% (w/v) BSA and 0.1% (v/v) Triton x-100 for 1 hr at RT. Staining with anti-PD-1 antibody (EH12.2H7, raised in mice, Biolegend) as well as a custom-made cocktail of anti-melanoma antibodies targeting tyrosinase, MelanA, and GP-100 (ab112231, ab118440, ab137078, all raised in rabbit, Abcam) was conducted in the same blocking buffer at 4 °C overnight. Slides were washed with 0.1% (v/v) Triton x-100 (3 × 15 min), before applying secondary antibodies (goat anti-mouse IgG STAR 635P and goat anti-rabbit IgG STAR 520 SXP, Abberior) in the blocking buffer at RT for 60 min. Slides were washed in 0.1% (v/v) Triton x-100 for 30 min, followed by TBS alone (1 × 5 min), before mounting in Mowiol mountant solution. Coverslips containing a 500 μm x 500 μm grid pattern were used to facilitate the identification of regions of interest identified from histology imaging.

### Fluorescence imaging

Large field-of-view diffraction-limited tile-scanning confocal imaging was conducted on a Leica SP5 upright confocal microscope. These images were used to identify the grid squares within the tumor regions identified in histology images and containing TILs (Figure 1B and 1C).

Stimulated emission depletion (STED) super-resolution fluorescence microscopy with a resolution of ~ 70 nm FWHM was conducted on a homebuilt microscope described previously [12]. Briefly, pulsed excitation at 635 nm and pulsed stimulating light at 750 nm are scanned along a fast axis using a 7.5 kHz resonant mirror and along a slow axis using a piezo stage. Fluorescence is collected between 550 and 615 nm through a pinhole of 0.7 Airy units, and detected using a Si avalanche photodiode. Image acquisition is controlled using a bespoke LabVIEW algorithm running on a field programmable gate array.

### Cluster quantification

**Figure 2:**
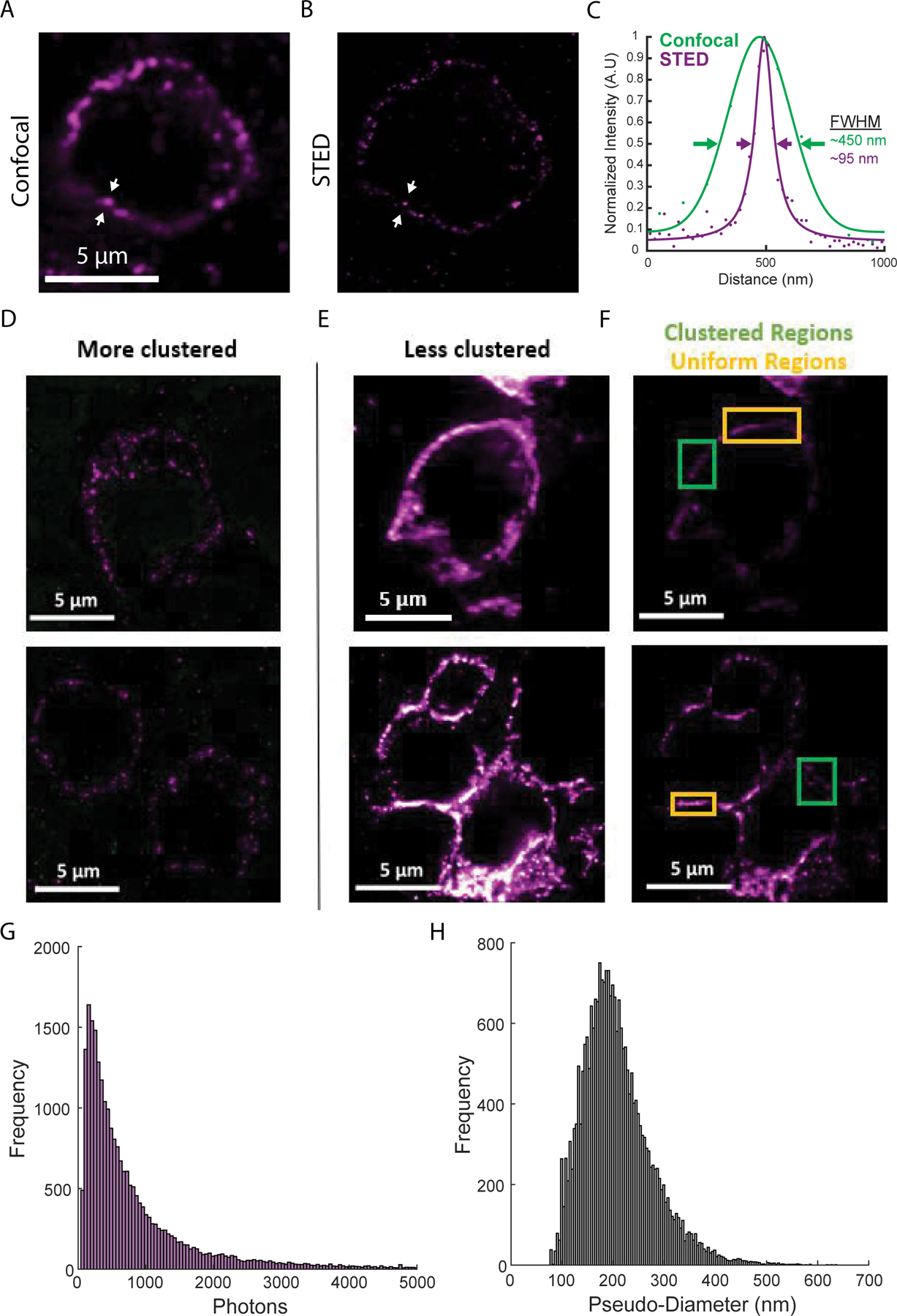
Super-resolution fluorescence imaging of PD-1 in the tumor microenvironment. (A) Diffraction limited confocal imaging of PD-1 in melanoma tissue. (B) STED super-resolution microscopy of the same region as (A). (C) Line profiles of the lines between the white arrows in (A, green) and (B, purple) show the resolution enhancement realized by STED microscopy. Solid lines show functional fits to data points. (D) Examples of cells with PD-1 are organized entirely in clusters. (E and F) Examples of cells with PD-1 are organized in clusters in some areas (green boxes) and with a uniform distribution in other areas (orange boxes). (E) and (F) show the same regions with adjusted color scales: 9–58 photons in (D and E); 20–150 photons in (F). (G) Histogram of cluster brightness as measured by integrated photons. (H) Histogram of cluster pseudo-diameter measured in nanometers. Histograms represent data from N > 24,300 clusters from more than 100 cells.

Intensity line profiles (Figure 2C) were determined for all pixels within 35 or 100 nm of the line (for STED and confocal images). Line profiles were fit to a 1D Lorentzian function, for STED images, or a 1D Gaussian function, for confocal images.

PD-1 clusters were identified using a wavelet product-based cluster segmentation algorithm described previously [13]. Cell regions were identified manually, to limit non-specific background staining. Raw images were Gaussian filtered (σ = 1.3 pixels), a rolling ball filter was used to remove the background (r = 50) and wavelet planes from k = 2–4 were considered. Cluster brightness was determined by integrating photon counts for each cluster. The pseudo-diameter was determined by fitting each cluster with an ellipse and taking the mean of the major and minor axis lengths.

## Results

To examine the subcellular distribution of PD-1 molecules in the tumor microenvironment, we obtained Formalin-Fixed Paraffin-Embedded (FFPE) tissue sections of matched primary and metastatic tumors from PD-L1-expressing melanoma patients. The first tissue section underwent standard clinical Hematoxylin-Eosin (HE) staining, allowing for the identification of tumor regions (Figure 1A). The adjacent tissue sections were fluorescently labeled using immunostaining (see methods). PD-1 was detected using a monoclonal antibody (EH12.2H7) and fluorescently labeled with a secondary antibody conjugated with the dye STAR 635P. Concentrations of both antibodies were titrated to achieve saturated labeling while minimizing non-specific background and any loss of resolution due to over-labeling. To more easily identify tumor regions, a cocktail of antibodies against melanoma tumor markers was fluorescently labeled with secondary antibodies tagged with STAR 520SXP. PD-1+ cells were easily identifiable in diffraction-limited images as ~ 10 μm diameter near circular cells, consistent with PD-1+ lymphocytes (Figure 1C–D). PD-1 labeling was further confirmed using tonsil tissue as a positive control (Supplemental Digital Content 1).

Typical super-resolution images are a few 10s of μm in size and can take minutes to hours to obtain. Tissue samples can be mm in size, making imaging of a complete section laborious. We developed an imaging workflow that first uses the histology images from adjacent tissue sections to identify tumor regions (Figure 1A) and then used low-magnification and low-resolution fluorescence imaging combined with gridded coverslips to identify these tumor regions for further super-resolution imaging (Figure 1B–C, see Methods for details). This process facilitated super-resolution imaging of regions in and around the tumor.

Using this workflow, we performed high-resolution immunofluorescence imaging of PD-1 in FFPE tissue sections from two melanoma patients. Diffraction-limited confocal microscopy images show apparent clustering of PD-1 on the cell membrane (Figure 2A); however, other areas appeared to show extended membrane labeling. Super-resolution STED microscopy (with a ~ four-fold increase in resolution to ~ 70 nm FWHM) revealed these extended structures were often composed of smaller clusters of PD-1 (Figure 2B). The resolution enhancement offered by super-resolution microscopy is more clearly demonstrated in line profiles across one of these clusters (Figure 2C).

We imaged over one hundred PD-1+ cells, observing phenotypes ranging from sparse, near resolution-limited nanometer-scale clusters (Figure 2D, ~ 85% of the membrane area) to a more uniform membrane labeling even at the higher resolutions obtained in this study (Figure 2E–F, ~ 15% of the membrane area). This clustering agrees with previous super-resolution studies of T cell receptors in mouse tissue sections [6], as well as PD-1 imaged on a glass-supported planar bilayer system [14]. These clusters exhibited a mean brightness of 968 ± 1,518 photons and a mean pseudo-diameter of 208 nm ± 68 nm (Figure G and H, mean ± STD of N > 24,300 clusters). There were no differences in cluster size between the primary tumor and lymph node metastasis and only a minor increase in cluster brightness for the primary tumor (Supplemental Digital Content 2). Together, these results demonstrate a large heterogeneity in the organization of PD-1 and represent early evidence that PD-1 can form clusters within the tumor microenvironment.

## Discussion

In this study, we set out to examine the nanometer-scale organization of PD-1 in the tumor microenvironment using super-resolution fluorescence microscopy. By immunolabeling FFPE tissue sections from excised human melanoma tumors and imaging using STED microscopy, we reveal the distribution of PD-1 with a resolution of ~ 70 nm FWHM. We demonstrate a broad range of PD-1 organizations, ranging from nanometer-scale clusters to more uniform membrane labeling (Figure 2D–F).

The clustering of surface receptors has been demonstrated for both innate [15] and adaptive immune cells [11], in cell culture [16,17] as well as in tissue samples [6] and for both activating [16–18] and inhibiting receptor molecules [19]. Our quantitative measurements of cluster size showing most clusters are between 80–300 nm in diameter are in good agreement with previous results for T cell receptor clustering [6,16,17]. The larger clusters (~ 300 nm - 900 nm) and more uniform regions of PD-1 shown in this study are reminiscent of the T cell receptor microclusters observed after antigen recognition both *in vitro*[16,17] and *in vivo* [6].

The clustering of immune receptors can play an important role in amplifying signaling by concentrating signaling partners, although a more complete understanding of the role receptor clustering plays in regulating immune signaling is still emerging. In T cells, PD-1 forms clusters with T cell receptors upon binding to PD-L1 in glass-supported lipid bilayers, leading to suppression of T cell activation. This suppression can be overridden by using a neutralizing anti-PD-L1 antibody [14]. Yet while PD-1 clustering and its importance in immune signaling have been demonstrated multiple times at such artificial immune interfaces in cell culture [20,21], there is limited evidence that such clusters form in the tumor microenvironment. To our knowledge, this study represents the first demonstration of PD-1 cluster formation in human tissue. Combining our observation that PD-1 clusters in the tumor microenvironment with others’ observations that PD-1 clustering suppresses T cell activation in cell culture [13], it is likely the presence of PD-1 clustering in the tumor microenvironment also leads to suppression of immune cell activation. Thus, levels of PD-1 clustering may correlate with ICI therapy outcomes. Future research is needed to connect the PD-1 clustering seen in this study with the suppression of T or other immune cell activation and to examine the effect of ICI therapy.

## Conclusion

Our application of super-resolution imaging of PD-1 in human melanoma tissue sections shows a broad range of phenotypes, ranging from nanometer-scale clusters to more uniform membrane labeling. Our demonstration of the presence of PD-1 clusters within the tumor microenvironment, combined with the previously demonstrated importance of such clusters in the suppression of T cell activation in cell systems, provides exciting new avenues for research in pathology and immunology. This imaging approach represents an important step towards bridging the gap between our understanding of immune receptor clustering from model cell systems and the more physiologically relevant tumor microenvironment.

## Supplementary Material

Supplement

## Abbreviations:

PD-1: Programmed Cell Death1
ICIs: Immune Checkpoint Inhibitors
PD-L1: PD-1 Ligand 1
FFPE: Formalin Fixed Paraffin-Embedded
TILs: Tumor Infiltrating Lymphocytes
STED: Stimulated Emission Depletion
