# Supplementary material for: Nanometer-scale distribution of PD-1 in the melanoma tumor microenvironment
Source: J Radiol Oncol. Author manuscript; Available in PMC 2023 Aug 3. (PMC10399701; doi:10.29328/journal.jro.1001048)
Supplement: Supplement [file NIHMS1918474-supplement-Supplement.docx]

**Supplemental Digital Content**

**Comerci CJ, McCarthy DG, Nosorati M, Kim KB, Kashani-Sabet M, Moerner W.E., Leong SP. Nanometer-scale Distribution of PD-1 in the Tumor Microenvironment**

**
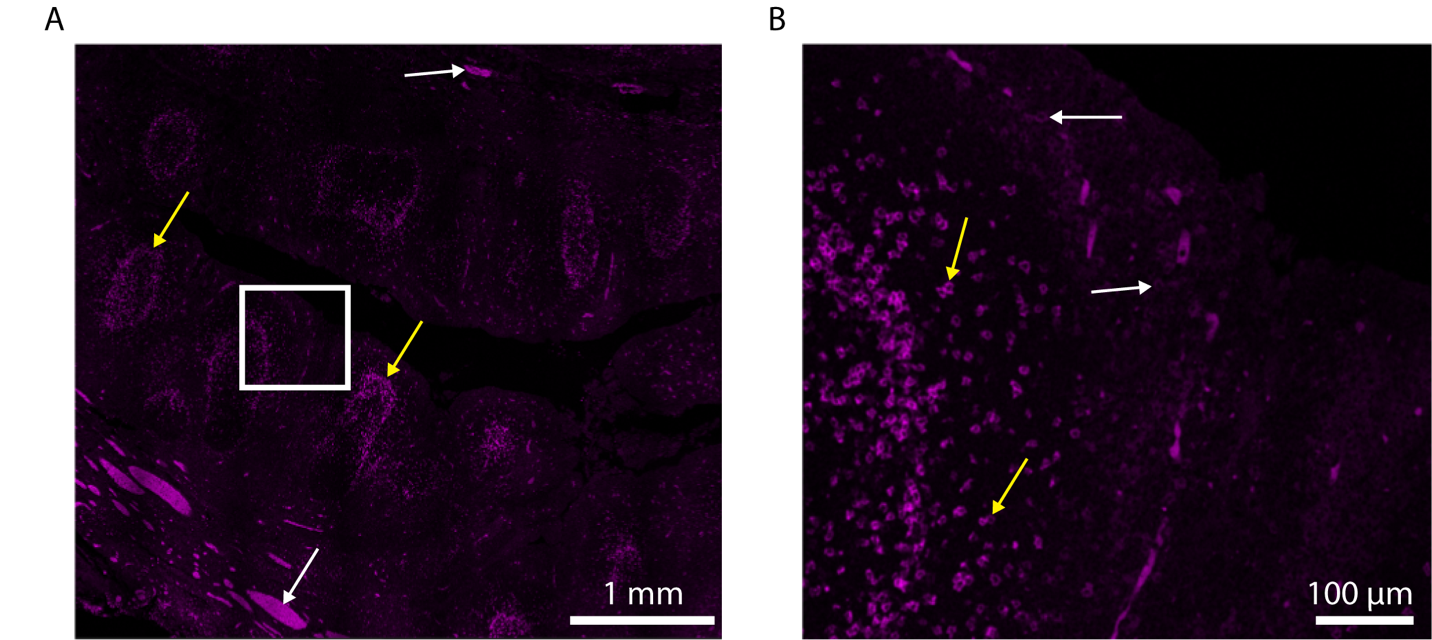
**

**Supplemental Digital Content 1: Single color images from diffraction limited confocal microscope of PD-1 in tonsil tissue. (A)** Image showing central areas of tonsillar crypt folds (examples marked with yellow arrows) with dense collection of strongly labeled PD-1^+^ cells and autofluorescence (examples marked with white arrows). The white box shows the area shown in (B). **(B)** Image showing T follicular helper cells (examples marked with yellow arrows) that are strongly labeled and interfollicular T cells (examples marked with white arrows) that are weakly labeled. Color-scale is 0-100 counts.


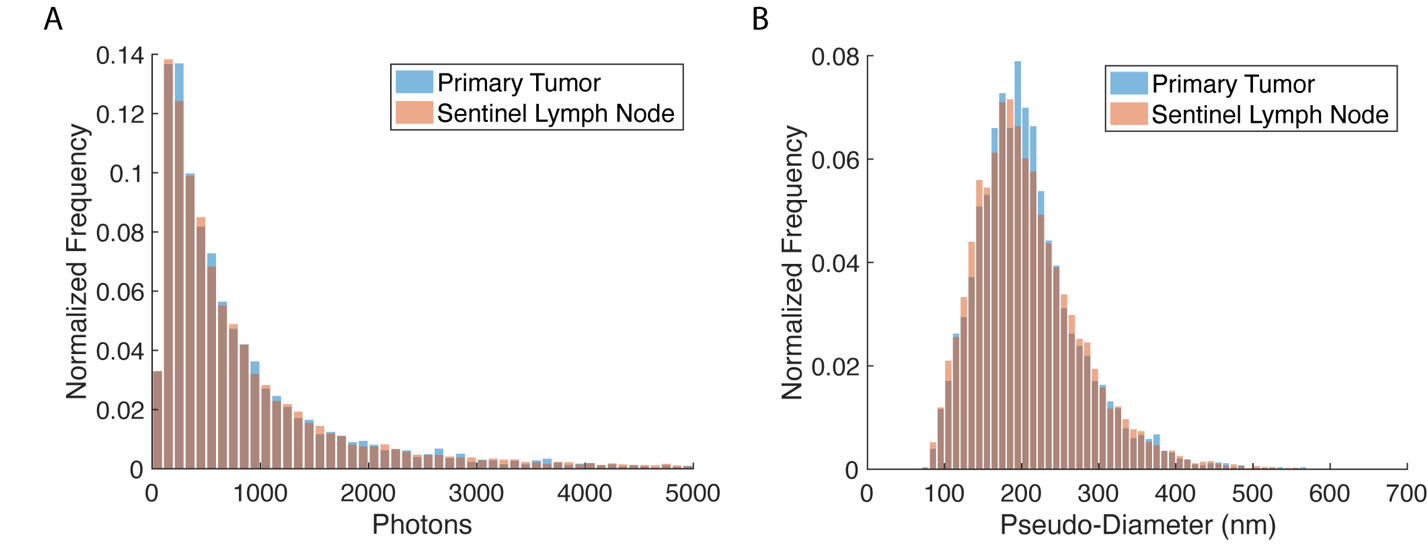


**Supplemental Digital Content 2: PD-1 cluster characteristics for primary melanoma tumor versus sentinel lymph node metastasis. (A)** Histogram of cluster brightness as measured by integrated photons for cells from the primary tumor (blue, 870 ± 1,084 photons) and lymph node metastasis (red, 996 ± 1,618 photons). Primary tumor clusters are marginally brighter than lymph node clusters (p = 0.04). **(B)** Histogram of cluster pseudo-diameters for cells from the primary tumor (blue, 208 ± 66 nm) and lymph node metastasis (red, 208 ± 69). Cluster size is not statistically different (p = 0.37). Values represent the mean ± STD of N > 5,000 clusters for the primary tumor and N > 19,000 clusters for the lymph node metastasis. Wilcoxon rank sum test used to determine differences in cluster distributions.
